# Supplementary figures and images for: The extent of algorithm aversion in decision-making situations with varying gravity
Source: PLoS One. 2023 Feb 21;18(2):e0278751. doi: 10.1371/journal.pone.0278751 (PMC9942970; doi:10.1371/journal.pone.0278751)

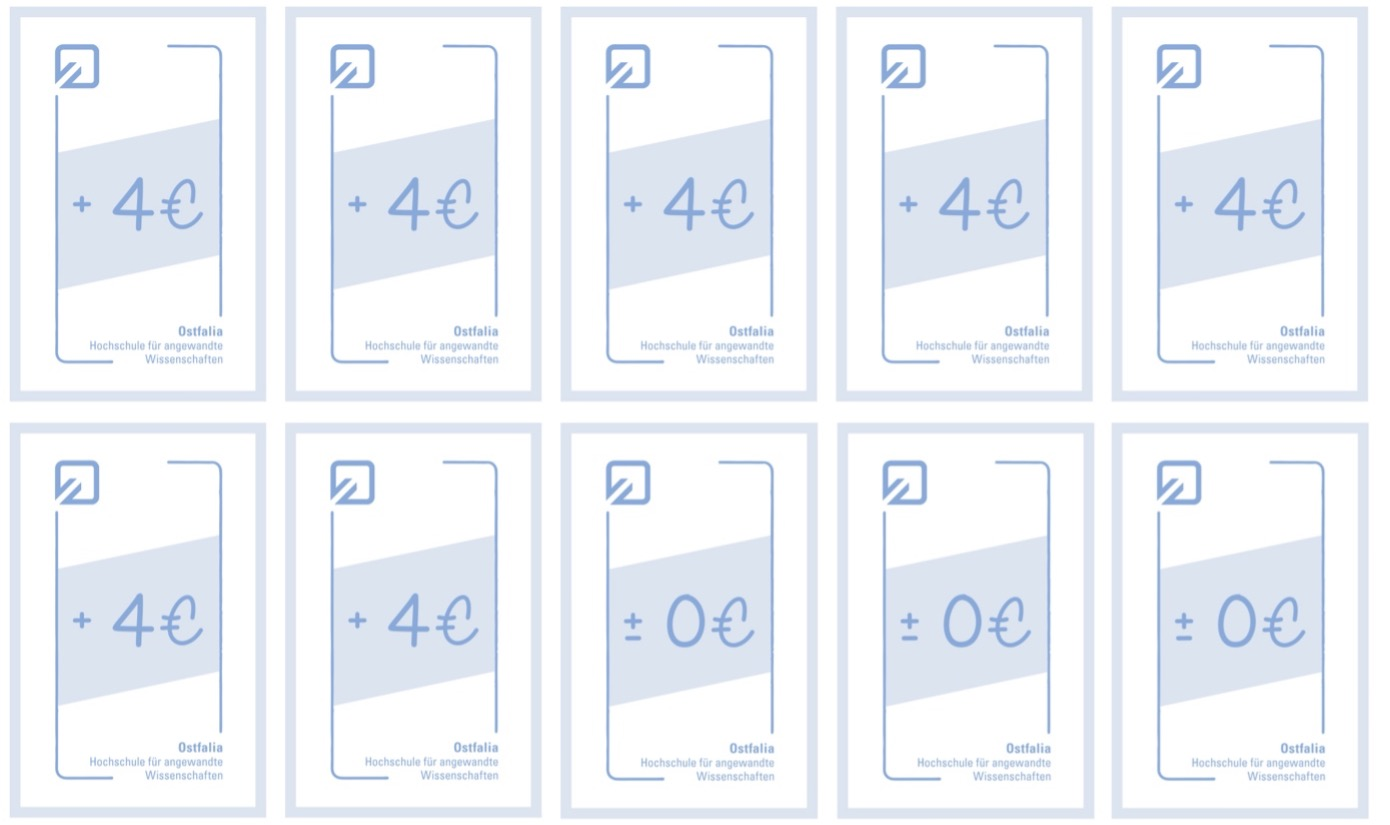

Supplement: S1 Fig — (TIF) [file pone.0278751.s001.tif]

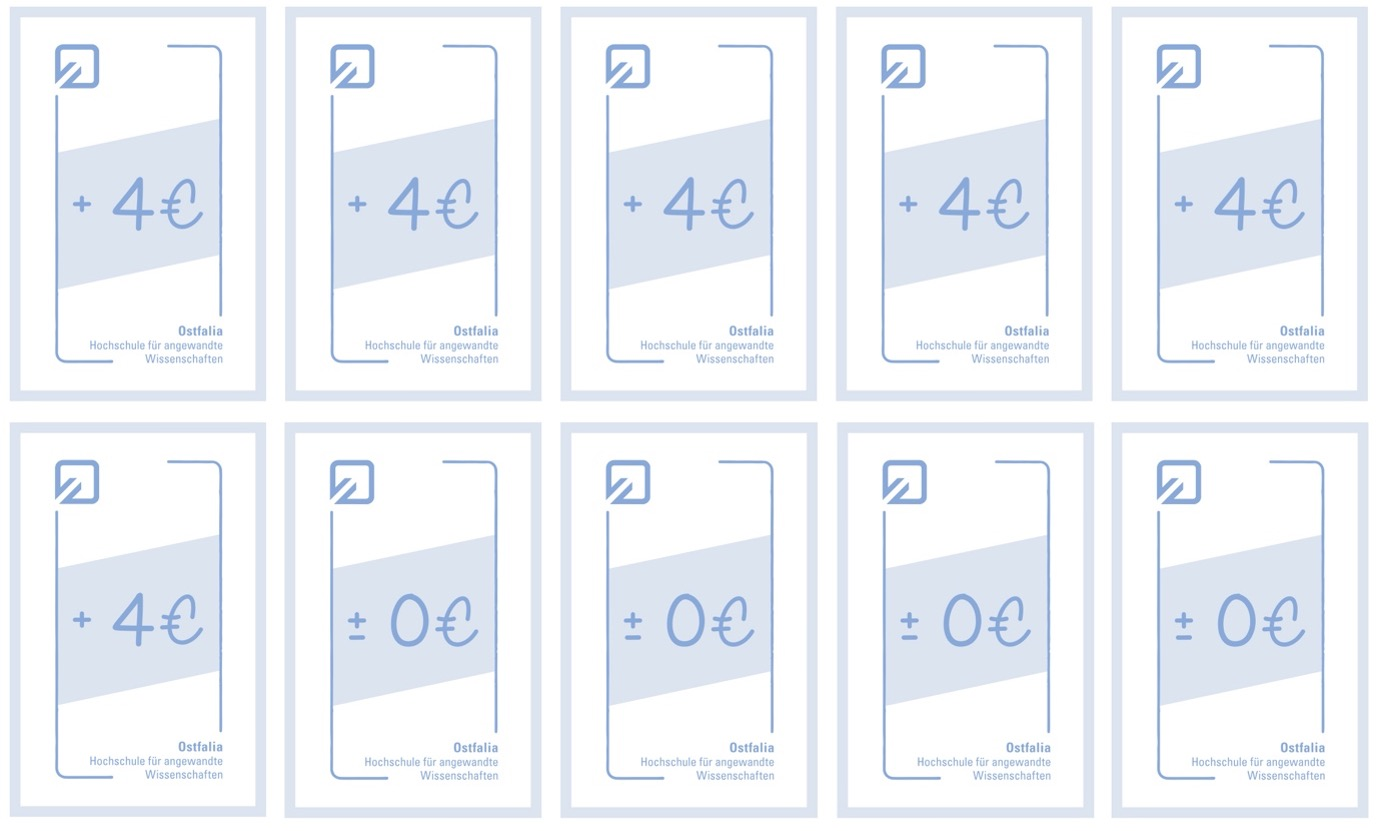

Supplement: S2 Fig — (TIF) [file pone.0278751.s002.tif]
