# Supplementary material for: The extent of algorithm aversion in decision-making situations with varying gravity
Source: PLoS One. 2023 Feb 21;18(2):e0278751. doi: 10.1371/journal.pone.0278751 (PMC9942970; doi:10.1371/journal.pone.0278751)
Supplement: S1 File — (DOCX) [file pone.0278751.s004.docx]

**The Extent of Algorithm Aversion in Decision-making
Situations with Varying Gravity**

**S1.** Instructions for the game

**The game**

You are a businessperson and have to decide whether you want a service you are offering for the first time carried out solely by an algorithm or solely by human experts. You are aware that the human experts carry out the task with a probability of success of 60%. You are also aware that the algorithm carries out the task with a probability of success of 70%.

**Procedure**

After reading the instructions and answering the test questions the decision-making situation is presented to you. This specifies the service which your company offers. First of all, you are asked to assess the gravity of the decision-making situation from the perspective of your customers. Then you decide whether the service should be carried out by human experts or by an algorithm.

**Payment**

You receive a show-up fee of €2 for taking part in the experiment. Apart from this, an additional payment of €4 is made if the service is carried out successfully.

**Information**

- Please remain quiet during the experiment
- Please do not look at your neighbor’s screen

Apart from a pen/pencil and a pocket calculator, **no** aids are permitted
