# Supplementary material for: The extent of algorithm aversion in decision-making situations with varying gravity
Source: PLoS One. 2023 Feb 21;18(2):e0278751. doi: 10.1371/journal.pone.0278751 (PMC9942970; doi:10.1371/journal.pone.0278751)
Supplement: S2 File — (DOCX) [file pone.0278751.s005.docx]

**The Extent of Algorithm Aversion in Decision-making
Situations with Varying Gravity**

**S2.** Test questions

**Test question 1:** Which alternatives are available to you to carry out the service?

1. I can provide the service myself or have it done by an algorithm.
2. I can provide the service myself or have it done by human experts.
3. I can have the service carried out via human experts or by an algorithm. *(correct)*

**Test question 2:** For how many newly-offered services do you need to make a choice?

1. None
2. One *(correct)*
3. Two

**Test question 3:** How much is the bonus payment for carrying out the task successfully?

1. €1
2. €2.50
3. €4 *(correct)*

**Test question 4:** How much is the bonus payment if you carry out the task wrongly?

1. -€2.50
2. €0 *(correct)*
3. €2.50
