# Supplementary material for: The extent of algorithm aversion in decision-making situations with varying gravity
Source: PLoS One. 2023 Feb 21;18(2):e0278751. doi: 10.1371/journal.pone.0278751 (PMC9942970; doi:10.1371/journal.pone.0278751)
Supplement: S3 File — (DOCX) [file pone.0278751.s006.docx]

**The Extent of Algorithm Aversion in Decision-making
Situations with Varying Gravity**

**S3.** Decision-making situations

**Decision-making situation 1:** Driving service

You are the manager of a public transport company and have to decide whether you want to transport your 100,000 passengers solely with autonomous vehicles (algorithm) or solely with vehicles with drivers (human experts). The task will be considered to have been successfully completed when all of your customers have reached their destination safely. In an extreme case, a wrong decision could mean the death of a passenger.

I choose: O Autonomous vehicles (algorithm)

O Drivers (human experts)

**Decision-making situation 2:** Evaluation of MRI scans

You are the manager of a large hospital and have to decide whether the MRI scans of your 100,000 patients with brain conditions should be assessed solely by a specialized computer program (algorithm) or solely by doctors (human experts). The task will be considered to have been successfully completed when all life-threatening symptoms are recognized immediately. In an extreme case, a wrong decision could mean the death of a patient.

I choose: O Specialized computer program (algorithm)

O Doctors (human experts)

**Decision-making situation 3:** Criminal case files

You are the head of a large law firm and have to decide whether the analysis of the case documents of your 100,000 clients should be carried out exclusively by a specialized computer program (algorithm) or solely by defense lawyers (human experts). The task will be considered to have been successfully completed when the penalties issued to your clients are below the national average. In an extreme case, a wrong decision could mean an unjustified long prison sentence for a client.

I choose: O Specialized computer program (algorithm)

O Defense lawyers (human experts)

**Decision-making situation 4:** Dating service

You are the manager of an online dating site and have to decide whether potential partners are suggested to your 100,000 customers solely by a specialized computer program (algorithm) or exclusively by trained staff (human experts). The task will be considered to have been successfully completed when you can improve the rating of your app in the App Store. For your customers, a wrong decision could lead to a date with a sub-optimal candidate.

I choose: O Specialized computer program (algorithm)

O Trained staff (human experts)

**Decision-making situation 5:** Selection of cooking recipes

You are the manager of an online food retailer and have to decide whether your 100,000 cooking boxes – with ingredients and recipes which are individually tailored to the customers – are put together solely by a specialized computer program (algorithm) or solely by trained staff (human experts). The task will be considered to have been successfully completed when you can increase the reorder rate as a key indicator of customer satisfaction. A wrong decision could mean that the customers don’t like their meal.

I choose: O Specialized computer program (algorithm)

O Trained staff (human experts)

**Decision-making situation 6:** Weather forecasts

You are the manager of a news site and have to decide whether your 100,000 daily weather forecasts for various cities are carried out solely by a specialized computer program (algorithm) or exclusively by experienced meteorologists (human experts). The task will be considered to have been successfully completed when the temperatures forecast the previous day do not diverge by more than 1 degree Celsius from the actual temperature. A wrong decision could mean that the readers of the forecasts do not dress suitably for the weather.

I choose: O Specialized computer program (algorithm)

O Experienced meteorologists (human experts)
