# Supplementary material for: The extent of algorithm aversion in decision-making situations with varying gravity
Source: PLoS One. 2023 Feb 21;18(2):e0278751. doi: 10.1371/journal.pone.0278751 (PMC9942970; doi:10.1371/journal.pone.0278751)
Supplement: S4 File — (DOCX) [file pone.0278751.s007.docx]

**The Extent of Algorithm Aversion in Decision-making
Situations with Varying Gravity**

**S4.** Determination of the random event with the aid of a lottery

**Fig A1.** Pack of cards in the selection of the algorithm.


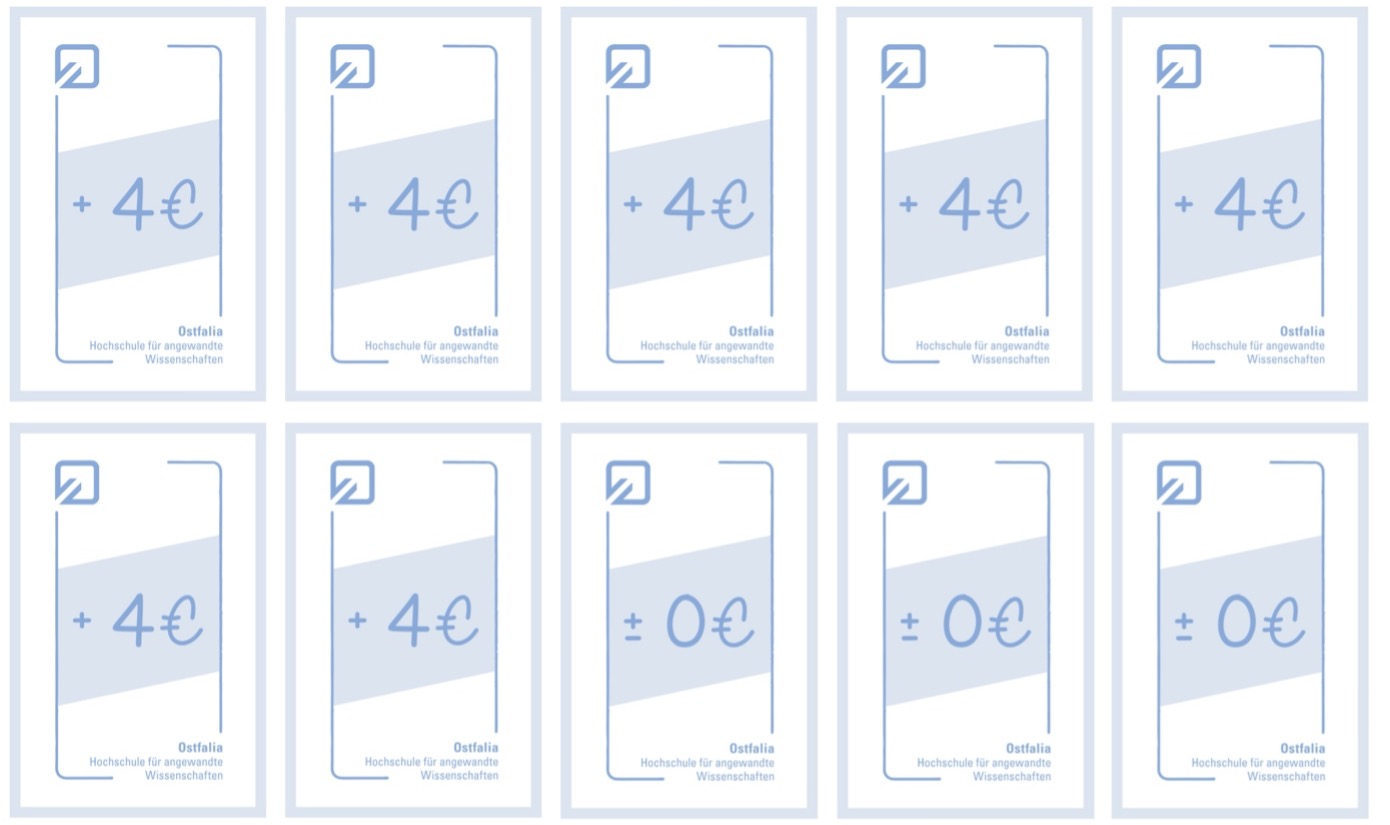


Pack of cards in the selection of the algorithm: seven cards with the event +€4 and three cards with the event €±0.

**Fig A2.** Pack of cards in the selection of the human expert.

**
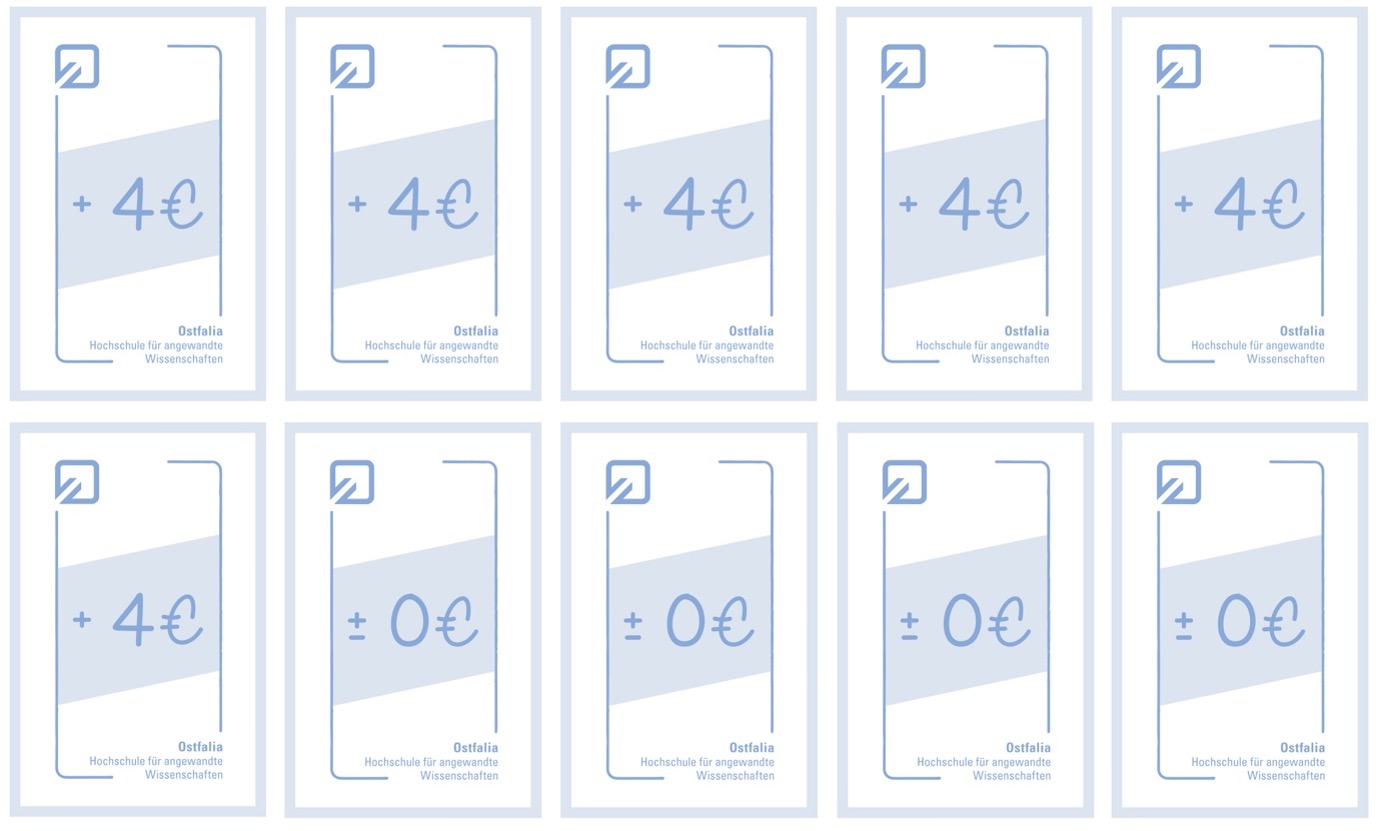
**

Pack of cards in the selection of the human expert: six cards with the event +€4 and four cards with the event €±0.
