# Supplementary material for: The extent of algorithm aversion in decision-making situations with varying gravity
Source: PLoS One. 2023 Feb 21;18(2):e0278751. doi: 10.1371/journal.pone.0278751 (PMC9942970; doi:10.1371/journal.pone.0278751)
Supplement: Attachment — Submitted filename: Framing and Algorithm Aversion - Rev Response.docx [file pone.0278751.s008.docx]

**Ref.: Manuscript ID PONE-D-21-39459 entitled "The Tragedy of Algorithm Aversion"**

Dear Dr. Schwieren,

We are very grateful that the reviewers approached our study with patience, diligence, and great expertise. We appreciate their helpful comments and suggestions. Unfortunately, in the end, it was not possible for us to run additional experiments. We have spent the last weeks working very hard to improve our manuscript with regard to the analyses and the links to the theory, as suggested by you. The proposed changes have been implemented as follows:

**Reviewer 1 (1):** The theory development relating the severity of the consequences of the decision and the presence of algorithm aversion needs more work. In the introduction, the authors briefly explain the relation mentioning the framing effect, but the relationship is not clear enough to the reader.

**Response:** Thank you for your suggestion, we have worked on our introduction and focused on our main concern. We hope the relationship is clear now (pages 2-3).

**Reviewer 1 (2):** In the derivation of hypothesis 3, the authors mentioned the model of bounded rationality, the cognitive limitations that humans suffer, and that great efforts are required when decisions have severe consequences. But why do decisions with more severe consequences induce less algorithm aversion, as Hypothesis 3 propose? Maybe the opposite could also be argued? Being the core of the paper, this relationship deserves a better explanation and discussion of the fundaments behind the possible behavior of decision-makers.

**Response:** To address this point in more detail, we inserted the following references:

- Lipman, B. L. (1995). Information Processing and Bounded Rationality: A Survey, *Canadian Journal of Economics*, 28(1), 42-67.
- Hoffrage, U., & Reimer, T. (2004). Models of bounded rationality: The approach of fast and frugal heuristics, *Management Revue*, 15(4), 437-459.
- Grüne‐Yanoff, T. (2007). Bounded Rationality. *Philosophy Compass*, 2(3), 534-563.

**Reviewer 1 (3):** Maybe the authors can derivate their theory based on how risky people perceived the task. Some risk definitions may help, like the one proposed by Kaplan and Garrick (1981), considering the "triplet" definition of risk as "scenarios, probabilities and consequences." Previous research has studied and suggests a higher presence of algorithm aversion in riskier circumstances (Dietvorst & Bharti, 2020; Grgic-Hlaca et al., 2019; Kawaguchi, 2020). For a general view, you can look at the high-level influencing factors of algorithm aversion described by Mahmud et al. (2022) in their literature review.

**Response:** Our focus is on the consequences of a decision and the frequency of algorithm aversion, but we added the paper by Mahmud et al. (2022), see page 2.

**Reviewer 1 (4):** A better explanation of why the six task were chosen would be useful related to the severity of the consequences. This may help considering as the authors mentioned, that in treatment B there is a larger range of severity evaluation, making some subjects assess the gravity of the decision as very high. Although authors ask how participants perceive the severity of the different tasks, finding significant differences between both treatment conditions, the tasks are in very different domains. Therefore, other phenomena could affect algorithm use rather than the severity of the consequences. Using control variables may help increase the robustness of the findings.

**Response:** We have explained the six tasks and their selection in more detail (page 8):

*The six decision-making situations that are part of this study were identified through a pre-test, in which additional decision situations were also presented from a literature analysis and brainstorming process. The final selection was made on the basis of three criteria: comprehensibility (do the subjects understand what this application area for algorithms is about?), familiarity (do the subjects know the application area from personal experience or from the media?), and scope (are the high and low scope decision situations actually evaluated as such?). The decision-making situations are selected in such a way that they are relevant in the literature and that the subjects should be familiar with them from public debates or from their own experience. In this way, it is easier for the subjects to immerse themselves in the respective context. The large number of different contexts ensures that other aspects from a specific frame than the severity of the consequences cannot distort the responses too much.*

**Reviewer 1 (5):** Regarding the experiment conducted, I would prefer a bigger sample than 143 participants considering the experiment design. If we consider that in each treatment, there are three tasks and, if I understand correctly is a between design study, every task has around 24 participants. More explanation on sample sizing with power calculations would be helpful.

**Response:** We planned it, but unfortunately it wasn't possible.

**Reviewer 1 (6):** The first paragraphs of the introduction focused on the definition of algorithm aversion. Although the discussion is valuable because I agree that different interpretations could be given to the concept "algorithm aversion" (Berger et al., 2020; Jussupow et al., 2020), I do not think it is the focus of this study. I suggest that the authors motivate in the introduction more directly to their main focus related to algorithm use and the connection between the consequences of a decision and the frequency of algorithm aversion.

**Response:** At your suggestion, we worked on our introduction and focused to algorithm use and the connection between the consequences of a decision and the frequency of algorithm aversion. We have now placed the general part in the chapter literature review (pages 4-7).

**Reviewer 1 (7):** Maybe another title for the paper could guide readers better to what the paper is about rather than "The Tragedy of Algorithm Aversion."

**Response:** We worked on a new title and settled on the following:

The Extent of Algorithm Aversion in Decision-making

Situations with Varying Gravity

**Reviewer 1 (8):** Providing supplementary materials such as data, surveys, analyses conducted, and preregistrations (if available) would be helpful. This may help promote further research in related topics, as well as promoting open science practices. Maybe supplementary material is available, and I was not aware.

**Response:** Thank you for your suggestion, we uploaded supplementary materials.

**Reviewer 2 (1):** Lack of control (I)

The experiment consists of 2 treatments, each treatment includes three "decision-making situations" (lines 119-130). This setup seems problematic as all six "situations" differ in many regards from each other (apart from the treatment effect "gravity of the consequences"). This relates to both differences between individual "situations" within one treatment as well as differences between individual "situations" across the two treatments and possible differences between treatments. Overall, these differences could provide alternative explanations for the observed effects that are not controlled for. In more detail:

Treatment A (possibly serious consequences) consists of the three decision making situations "Autonomous driving", "Evaluation of MRI scans" and "The assessment of criminal case files", whereas treatment B (no serious consequences) consists of "Dating service", "Selection of recipes" and "Drawing up weather forecasts". The situational descriptions include many factors that could influence the decision of participants and that are often associated with algorithm aversion in the literature.

For the description of the situations of treatment A this, for example, relates to factors such as morality and ethics (e.g. ethical questions related to decisions of autonomous vehicles in potential accident situations, morality questions in delegating criminal investigations). From the algorithm aversion literature it is also well understood that people in particular tend to distrust algorithms for medical diagnosis (here: MRI scan). In this situation, participants might in addition find it disturbing (or "immoral") that the decision to rely on an algorithm is not made by a doctor but by the manager of the hospital (lines 511ff.). For the decision situations in treatment B these factors seem to be of less importance.

Two of the situations of treatment B (dating services and receipts) appear to be more subjective in nature as compared to the situations of treatment A (in particular with regard to autonomous driving, but to a lesser extend also with regard to the other two "situations"). This also constitutes a difference to the less subjective third situation of treatment B (Weather forecast). This third "situation" of treatment B furthermore appears to differ in the degree of complexity. In addition, for weather forecasts participants might "expect" algorithms being involved. Overall, it seems that participants could potentially be more familiar with the use of algorithms in all three situations of treatment B. In the real world, algorithms are frequently used in such contexts already and real life experience of participants seems more probable here. Complexity, subjectivity and familiarity are all factors identified in the literature to affect algorithm aversion. This list is not exhaustive and more factors not controlled for might play a role.

**Response:** This is also a good comment, which we have added to the limitations of the study. However, our regression analysis shows that the core result of the study is unaffected by all these aspects. Certainly, driving a car may be relatively insignificant to one subject, and cooking recipes very significant to another subject. The possible associations that the tasks evoke in the subjects are innumerable and immeasurable and therefore cannot even be discussed in detail in this study. However, all the mentioned points are not part of the regression analysis. There, only the correlation between algorithm aversion and the subjectively perceived (!) significance is mapped, and the result is as highly significant effect.

**Reviewer 2 (2):** Lack of control (II) - The authors explain that "The decision-making situations are selected in such a way that the subjects should be familiar with them from public debates or from their own experience. In this way, it is easier for the subjects to immerse themselves in the respective context." (lines 134-137). Subjects will indeed very likely be familiar with the situation or context of the decisions from the real world (though to different degrees, as explained above). But this results in a loss of control over the experiment and therefore also appears problematic:

The study defines a clear superiority of algorithms for all six "situations" identically (70% probability of success of the algorithm compared to 60% probability for human experts, lines 139-141). According to the definition of the authors, algorithm aversion only exists if subjects chose human experts despite the superior performance of algorithms. It appears problematic that better performance might not be given for the chosen situations, at least with regard to participants` experience and perceptions of the real world. For autonomous shuttle busses, for example, technological development is still at an early stage and the real world performance is often perceived as being (still) insufficient or at least poorer than that of a human operator. Choosing a human operator might thus be the result of this real-world experience or knowledge and not of algorithm aversion.

**Response:** The probabilities of success are an aspect that we have discussed at length in our team. Our main conclusion is that in a framing experiment, only one aspect may be changed between situations, and here that is the context of the decision. Success rates must remain unchanged, otherwise we would not know where differences in subjects' responses come from. We therefore included this point in the limitations.

**Reviewer 2 (3):** With regard to the chosen (student) subject pool this problem seems to be of particular relevance. Some of the decision situations seem to be directly linked to the content of the study programs of participants (autonomous driving/ health diagnosis). 60 subjects (42.0%) study at the Faculty of Vehicle Technology, and 18 subjects (12.6%) at the Faculty of Health Care (lines 201 and 202).

**Response:** True. This is a typical limitation of economic experiments with students as subjects. To minimize potential bias, we ensured an even distribution of study programs and genders and documented this in the Supplementary Material. In addition, we checked the influence of study program and gender on both the assessment of the gravity of consequences and the human versus algorithm decision and found no significant influence.

|  | **Faculty** | | | **Total** |
| --- | --- | --- | --- | --- |
|  | Vehicle Technology | Health Care | Business |  |
| A1 - Autonomous driving | 8 | 5 | 12 | **25** |
| A2 - MRI scans | 12 | 3 | 9 | **24** |
| A3 - Criminal case files | 10 | 4 | 8 | **22** |
| B1 - Dating service | 13 | 2 | 9 | **24** |
| B2 - Selection of recipes | 7 | 2 | 14 | **23** |
| B3 - Weather forecasts | 10 | 2 | 13 | **25** |
|  |  |  |  |  |
| Total | 60 | 18 | 65 | 143 |

|  | **Sex** | | | **Total** |
| --- | --- | --- | --- | --- |
|  | Male | Female | Other |  |
| A1 - Autonomous driving | 16 | 9 | 0 | **25** |
| A2 - MRI scans | 15 | 9 | 0 | **24** |
| A3 - Criminal case files | 14 | 8 | 0 | **22** |
| B1 - Dating service | 16 | 8 | 0 | **24** |
| B2 - Selection of recipes | 14 | 8 | 1 | **23** |
| B3 - Weather forecasts | 16 | 8 | 1 | **25** |
|  |  |  |  |  |
| Total | 91 | 50 | 2 | 143 |

**Reviewer 2 (4) :** Problems related to the incentive structure. With the chosen experimental design, it remains somewhat unclear whether subjects base their decision on the situational description or on the performance factor/ the success probabilities provided in the introduction of the experiment. Participants could either understand the missing link between incentive scheme and situational descriptions. In this case it seems probable that some participants understand the game as a choice between two lotteries and decide solely based on the probabilities of the lotteries ignoring the context completely. Or they could base their decisions on the situational descriptions. In this case it would seem likely that participants are influenced by real world experience and not only (if at all) by the success probabilities of the card decks provided. The perceived real world performance might contradict with the probabilities provided. The heterogeneous background of the participants (different study programs that are related to the scenarios) might also play a role here.

**Response:** That is exactly how it is. A rational utility maximizer (homo economicus) would only make decisions on the basis of the lotteries. All decisions would then have to be made in favor of the higher expected value of the remuneration, i.e. in favor of the algorithm. Obviously, however, this does not turn out to be the case in our results. Subjects decide not only based on the probabilities of success, but also based on the scenario, and this is exactly what we wanted to show with this study. Since the incentive structure is identical in all six scenarios, which is the core of our framing approach, we could prove beyond doubt that the context and the consequences of the decision have a massive impact on algorithm aversion.

**Reviewer 2 (5):** The lotteries are implemented with the help of physical card decks (lines 214/215). This non-digital implementation of the lotteries might further support the "decoupling" of incentive and situational description in this particular context (algorithms).

**Response:** In our eyes, implementing the lottery through cards is a very good way to ensure that subjects see that there is a true random process. If the computer simply displayed a result, subjects would not have this assurance.

**Reviewer 2 (6):** Probabilities are "made up" and not created within the experiment.

**Response:** This is also a very good point, which we have added to the limitations. It was important for us to conduct this research in the context of important actual application areas of algorithms in order to gain insights for these highly relevant topics. Of course, it is not possible from a research ethics point of view (and also from a criminal law point of view) to let the subjects experience for themselves the success rates of an autonomous vehicle or a cancer diagnosis. Therefore, we need to abstract at this point and this design seemed to be a suitable way to do so. From our point of view, the crucial point to enable a comparison between scenarios is that the performance difference between algorithm and human is kept constant.

**Reviewer 2 (7):** It should be noted that the success probabilities are explained in the introduction of the experiment, but not in the situational descriptions. This might affect the salience of this information.

**Response:** The indication of the probabilities of success in the introduction of the experiment (in the instructions) is a deliberate design decision, so that the described situation may have an effect on the subjects and the decision situation can be described briefly and concisely. The survey for our experiment took place in the laboratory in presence. Subjects took their seats and received the instructions on printed paper (see Appendix 1 of the manuscript). In the instructions, the probabilities of success for carrying out the task by the human expert successfully and for carrying out the task by the algorithm successfully are given. Since the printed instructions are available to the subjects throughout the processing of the experiment, the success probabilities can be reviewed at any time. Thus, the information about the success probabilities is always available to the subjects.

**Reviewer 2 (8):** The incentive scheme does not take differences in gravity into account.

**Response:** Again, this is a deliberate design decision to fit our chosen framing approach. We bring the same mathematical ratios to the probabilities of success (60% execution of a human expert and 70% algorithmic execution) into a different situational context. Only the different situation descriptions - with the same success probabilities - constitute the framing approach. We change only one aspect - that is the situation description - and keep the rest constant so that it is clear where the differences in decision behavior arise from (different situations).

**Reviewer 2 (9):** Lines 336-337: "The differing consequences of the decision-making situations do not affect the subjects themselves, but possibly have implications for third parties." Such implications for third parties are also purely hypothetical and outside the incentive scheme of the experiment.

**Response:** This is exactly how vignette studies work. In our study, from a research ethics point of view, we surely cannot expose the subjects to the serious consequences (autonomous driving - death; evaluation of MRI scans - malpractice/death; evaluation of case files - long prison sentence) that we are investigating in our exciting research question. Subjects are faced with the decision situations and must decide. Vignette studies are the only way to shed light on questions such as ours. Subjects know the impact of not successfully performing a task and know the impact on their compensation.

**Reviewer 2 (10):** The description and analysis of the data seems somewhat incomplete: Line 203: How many participants for the individual scenarios?

**Reviewer 2 (11):** Lines 226ff. Gravity check: no information provided on how participants assessed the gravity of the six individual scenarios. The scenarios within a treatment are added up without any further analysis. Differences between the scenarios could also (partially) explain why "subjects perceive the gravity of the decision-making situations significantly differently".

**Response (10+11):** We added the number of subjects per decision situation/situation description in the Results section:

*Of the participants, 71 subjects are in a decision-making situation which has been assigned to Treatment A (autonomous driving 25 subjects, evaluation of MRI scans 24 subjects, the assessment of criminal case files 22 subjects), while 72 subjects are presented with a decision-making situation which has been assigned to Treatment B (dating service 24 subjects, selection of recipes 23 subjects, drawing up weather forecasts 25 subjects).*

Furthermore, we now present an additional table showing all six decision situations individually with the number of subjects who went through them and the associated mean value of the gravity of a single situation description:

*Even though the six different decision situations differ considerably from each other in their context, they are also similar in the assessment of their gravity when viewed individually. The possible consequences of each decision situation from treatment A are estimated by the subjects to be much more serious than the possible consequences of each decision situation from treatment B (see Table 4).*

***Table 4.*** *Overview of the mean values of the gravity per decision situation.*

| *Situation description*  *per treatment* | *#* | *Mean value of the gravity* |
| --- | --- | --- |
| *A-1 Autonomous driving* | *25* | *8.88* |
| *A-2 Evaluation of MRI scans* | *24* | *9.42* |
| *A-3 Criminal case files* | *22* | *8.68* |
| *B-1 - Dating service* | *24* | *6.33* |
| *B-2 Selection of recipes* | *23* | *7.87* |
| *B-3 Weather forecasts* | *25* | *5.52* |

We have also updated former Table 4 (now Table 5) to show the full listing of the six situation descriptions split between the two treatments:

***Table 5.*** *Decisions for and against the algorithm.*

|  |  |  | *Decisions for*  *the algorithm* | | *Decisions against*  *the algorithm* | |
| --- | --- | --- | --- | --- | --- | --- |
|  |  | *Total* | *#* | *Percent* | *#* | *Percent* |
| ***Treatment A*** | ***(serious)*** | ***71*** | ***36*** | ***50.70%*** | ***35*** | ***49.30%*** |
| *A-1 Autonomous driving* | | *25* | *13* | *52.00%* | *12* | *48.00%* |
| *A-2 Evaluation of MRI scans* | | *24* | *13* | *54.17%* | *11* | *45.83%* |
| *A-3 Criminal case files* | | *22* | *10* | *45.45%* | *12* | *54.55%* |
| ***Treatment B*** | ***(not serious)*** | ***72*** | ***51*** | ***70.83%*** | ***21*** | ***29.17%*** |
| *B-1 Dating service* | | *24* | *18* | *75.00%* | *6* | *25.00%* |
| *B-2 Selection of recipes* | | *23* | *14* | *60.87%* | *9* | *39.13%* |
| *B-3 Weather forecasts* | | *25* | *19* | *76.00%* | *6* | *24.00%* |
| ***Σ*** | ***(total)*** | ***143*** | ***87*** | ***60.84%*** | ***56*** | ***39.16%*** |

**Reviewer 2 (12):** Analyze for possible differences resulting from different backgrounds (study programs)

**Response:** We analyzed our collected data and results in advance for significant differences between the study programs of the participating subjects (automotive engineering, health care, and business) in decision-making behavior. Since no significant differences emerged here, we refrained from mentioning them in the manuscript. If required, we are happy to mention in the manuscript that there were no significant differences between the subjects' study programs.

**Reviewer 2 (13):** Analyze for possible gender effects (minor comment)

**Response:** Here, the same applies from our response to your previous comment. We analyzed our collected data and results in advance for gender effects. Again, no significant differences emerged, which is why we refrained from mentioning them in the manuscript.

**Reviewer 2 (14):** Discuss the results of the manipulation check in more detail. Did manipulation really work properly for all six situations (e.g. recipes)?

**Response:** From our point of view, we already go into detail about the results of the manipulation check, and we show Table 3 and Figure 2 to support the results. Please find our explanations from the manuscript below:

*The results of the manipulation check show that the subjects perceive the gravity of the decision-making situations significantly differently (Table 3 and Fig 2). In the decision-making situations with serious consequences (Treatment A), the average of the perceived gravity is 9.0 with a standard deviation of 1.37. In the evaluation of the gravity of the decision-making situations of Treatment B on the other hand, there is an average of 6.54 with a standard deviation of 2.53.*

*Table 3. Evaluation of gravity in Treatments A and B.*

*The graphical analysis also shows that overall, the subjects assess the gravity in Treatment A to be more serious than in Treatment B. In a direct comparison of the violin plots, however, it is obvious that there is a larger range than in Treatment A, because some subjects also assess the gravity as very high in Treatment B (Fig 2).*

*The Wilcoxon rank-sum test (Mann-Whitney U test) (cf. Wilcoxon, 1945; Mann & Whitney, 1947) shows that the gravity of the decision-making situations in Treatment A is assessed as being significantly higher than that of the decision-making situations in Treatment B (z = 6.689; p = 0.000).*

Nevertheless, we have taken up your comment: The new Table 4 we inserted also shows in more detail that the manipulation check had an effect on the subjects for all six situation descriptions of the two treatments (for recipes as well). The mean values of the gravity from treatment A are all higher than the mean values of the gravity from treatment B, which means that the gravity in the treatment with potentially serious consequences were estimated to be higher than the gravity in the treatment with no serious consequences.

**Reviewer 2 (15):** The authors argue that the "decisive advantage of a framing approach is that the influence of a factor can be clearly identified. There is only one difference between the decision-making situations in Treatment A and Treatment B: the gravity of the possible consequences." (lines 306 -308). I cannot fully agree with this statement. All six situations differ from each other by more than one factor. Also, all three situations of treatment A taken together seem to differ by more than one factor from the situations of treatment B taken together. Some of these factors have been identified in previous literature as being particularly linked to algorithm aversion. In addition, the incentive scheme is not fully convincing. As a result, it to some degree remains unclear whether the observed differences are the result of the treatment effect or of other (uncontrolled for) differences or of real world experience. Some of these problems could possibly be addressed by a more in depth analysis of the data (the regression analysis described in lines 277ff. indicates a promising direction as it does not rely on the aggregation of scenarios into treatments), whereas some of the limitations seem inherent to the experimental design and should, at least, be discussed as such.

**Response:** Thank you for your detailed comment on this issue. From our point of view, a more detailed analysis per frame does not make sense due to the small number of subjects who went through a decision situation. Unfortunately, it was not possible for us to conduct another experiment to obtain more data for a robust analysis of the six individual description situations.

**Reviewer 2 (16):** Line 41: for a very recent systematic literature review also see: Hasan Mahmud, A.K.M. Najmul Islam, Syed Ishtiaque Ahmed, Kari Smolander, What influences algorithmic decision-making? A systematic literature review on algorithm aversion, Technological Forecasting and Social Change, Volume 175, 2022, 121390.

**Response:** Thank you for referencing this recent overview. We have included the study and recommend it in the same place where we list other literature reviews on Algorithm Aversion.

**Reviewer 2 (17):** Line 64/table 1: Sometimes a definition is provided that is only an indirect quote of a definition already provided elsewhere in the table. The added value of doing so remains somewhat unclear.

**Response:** We see the added value in this overview to bring clarity about the different understandings of the term 'Algorithm Aversion' by a variety of authors. Although some authors refer to the definition of other authors, the understanding of the term 'Algorithm Aversion' in the scientific discourse is to some extent very different. Some of these definitions may be flawed, immature, or based on a misunderstanding of previous studies, and it is for this specific reason that we feel it is of particular importance to provide clarity in the definition of the term 'Algorithm Aversion' in the scientific discussion. When the term 'Algorithm Aversion' is used in scientific literature, it should be based on the same understanding so that research can advance to results for understanding this phenomenon.

**Reviewer 2 (18):** Lines 93-94: More careful formulation suggested with regard to normative recommendations in particular when other aspects (e.g. ethical considerations) may also be of importance.

**Response:** We understand the comment, which is why we are happy to use a more careful formulation at this point:

*In these serious cases, it can be expected that people tend to think more thoroughly about what to do in order to make a reasonable decision. (now in lines 53-54)*

**Reviewer 2 (19):** Test questions: Explain what happens if someone answers test questions incorrectly (I assume that question needs to be answered again, but this is not explicitly mentioned in the manuscript).

**Response:** We understand your comment and see the added value for the reader. For this reason, we have included some explanations in the manuscript on the handling and procedure of our test questions so that there is no ambiguity for the reader:

*Before the experiment begins, all participants have to answer test questions (Appendix 2). They have a maximum of two attempts at this. Participants who answer the test questions incorrectly twice are disregarded in the analysis, as the data should not be distorted by subjects who have misunderstood the task.*

We appreciate your time and effort and would like to thank the reviewers for their suggestions which have helped us improve our work. We are glad to see how our manuscript has developed and hope that you find it suitable for publication. Should you find our paper in need of additional correction, we are of course prepared to make further changes.
