# Supplementary material for: The extent of algorithm aversion in decision-making situations with varying gravity
Source: PLoS One. 2023 Feb 21;18(2):e0278751. doi: 10.1371/journal.pone.0278751 (PMC9942970; doi:10.1371/journal.pone.0278751)
Supplement: Attachment — Submitted filename: Response to Reviewers.docx [file pone.0278751.s009.docx]

**Ref.: Manuscript ID PONE-D-21-39459R1 entitled "The Tragedy of Algorithm Aversion"**

Dear Dr. Schwieren,

We are very grateful that you and the reviewers approached our study with patience, diligence, and great expertise. We appreciate both you and the reviewers for your helpful comments and suggestions. Again, we have spent the last weeks working very hard to improve our manuscript with regard to the analyses and the links to the theory, as suggested by you. The proposed changes have been implemented as follows:

**Editor (1):** Even though I see that adding more data is impossible at this stage (it would have been a major improvement), being more clear in the description of the theory behind the hypotheses is, in my view, key. Adding literature is one thing, that is certainly an improvement, but considering this literature thoroughly would improve the paper even more.

**Response:** Thank you very much for this advice. We have made substantial revisions in the "Methods and experimental design" section and have more clearly described the derivation of hypotheses 2 and 3 (the core of this paper) to capture the underlying theories more thoroughly.

**Editor (2):** The same holds for the discussion of design choices that might not be considered ideal in hindsight. Every experiment has design choices that, after the fact, are considered not ideal. However, if there are ways to deal with that in the data analysis - if only in being very careful with respect to causal language - is warranted, if it is impossible to add to the data collection.

**Response:** We have expanded our data analysis in the "Results" section to provide greater insight into our findings. In doing so, we now also address the six individual decision situations in terms of the perceived gravity of the situation and the subjects' delegation decision. We use cluster analysis to explain in more detail how we group decision situations into not serious and serious. In doing so, we have been objective and careful with causal language, hoping to provide better insight into the data and the subjects' behavior. We are also aware that there are limitations in any study and design decisions may prove to be not ideal in hindsight. We address this fact of our study in the "Discussion" section and specifically identify these limitations as a potential weakness of our study in the "Limitations and Future Research" subsection.

**Editor (3):** I also see some contradictions in the argumentation of the authors with respect to the vignettes. It is clearly NOT the case that the only thing that changes is gravity - it is always context AND gravity that changes, and thus, the results are less clear than the authors claim.

I can imagine that the authors might consider this too much change to the paper, but even though Plos One differs in publication criteria from other outlets, it does have a strong commitment to clear and correct data analysis and interpretation of the results - including to be careful not to oversell.

**Response:** As previously mentioned, we greatly expanded the scope of our data analysis to provide better insight into subjects' decisions. We have removed the reference to vignette studies that we made in the 1st revision. We also address in the "Results" section that not only does the gravity of a scenario change, but the change in gravity is contextually related to the scenario being described. Based on the described context, subjects make a gravity rating. Thus, the gravity level and context change between scenarios.

**Reviewer 1 (1):** I want to thank the authors for their work, but I do not think the current status of the paper meets the publication standard required. Reading the responses to my comments and the revised manuscript, I realize that the authors have not adequately addressed my major concerns. Particularly my major concerns regarding the theory development is not well explained in the introduction, and only adding references to the derivation of hypothesis 3 is not enough to clarify the theory relationship between severity of the consequences and algorithm aversion. Also, I think that 143 participants is a very small sample, as I mentioned before, and probably the experiment is underpowered. Unfortunately, the authors were not able to conduct new experiments. Therefore, I think this paper requires significant rework and especially conducting more experiments to get published.

**Response:** Thank you very much for your comment. We have used the last weeks to improve both the derivation of the hypotheses on the relationship between the gravity of a decision and the decision made, as well as in the data analysis to provide better insights into the results of our study. We significantly extended the derivation of hypotheses 2 and 3 (core of this study) and described in more detail why a relationship between the gravity of a decision and the decision made (algorithm aversion) is hypothesized. We also made additions to the theory development in the introduction of the study.

Unfortunately, it is not possible to conduct further experiments. However, to address this point we have done further analysis in the "Results" section to support our findings using cluster analysis and others to be more clear in describing our results. In addition, we clearly state in the limitations that the sample size is small with 143 subjects.

**Reviewer 2 (1):** The authors have considered some of the comments and have improved the paper considerably. However, this does not fully apply to the main problem I have seen (lack of control/ risk of spurious results). In contrast to the authors' explanation, I am still not fully convinced that the design allows for a clear proof of causality with respect to the correlation between gravity and algorithm aversion.

A causal relationship between two variables exists if a variation in the independent variable results in a variation in the dependent variable, keeping all other things equal (ceteris paribus). As explained in my previous comments, the scenarios (or treatments) are quite different from each other, they differ in many aspects. The authors have included a sentence addressing this difference now. But they, in my view, still do not recognize that as a result of these differences the relationship between the two variables could be spurious/ due to changes in a third variable.

**Response:** We performed a cluster analysis and included the results in our study. The decision scenarios are compared in different clusters. The cluster of the gravity of a decision situation is shown to be highly significant in contrast to other clusters. In addition, we added a confidence interval to the evaluation of our main research question and included the influence of numerous other independent (demographic) variables on algorithm aversion to check for spurious results.

Nevertheless, we understand that our study is subject to some limitations and address in the "Discussion" section that our chosen study design poses risks in terms of causality. In this way, we hope to provide guidance to the reader in understanding our results and their limitations.

**Reviewer 2 (2):** In the reply letter, the authors (if understood correctly) imply that their experiment constitutes a vignette study (author response to Reviewer 2 comment 9). I also do not find this argument convincing. While I agree with the authors that a vignette design could proof useful to answer the RQ, I do not believe that such a design has been implemented (the term "vignette" does also not appear in the paper). Vignette surveys (factorial surveys) include a description of a situation, consisting of a systematic combination of characteristics ("dimensions") and a systematic variation of these dimensions ("levels"). Through experimental variation unconfounded effects of the dimensions/ factors can then be estimated. The experiment on hand does, however, not vary the dimension "gravity" within one scenario (e.g. MRT scans for live threatening diagnosis vs. MRT scans for less severe diagnosis; weather forecast with possible live threatening consequences, e.g. for sailors vs. weather forecast with less severe consequences such as a beach day which is cancelled), but compares across different scenarios. This difference to the standard vignette experimental design is (again) problematic with regard to causality.

As explained earlier, this problem seems inherent to the experimental design to me. I believe that the experiment can still add value to the literature, but that this limitation (if correctly observed and shared by the other reviewer) is still not addressed sufficiently in the paper.

**Response:** We have removed the reference to the vignette study that we added as part of the 1st revision. However, we have taken up the point you describe in the "Discussion" section and mention in "Limitations and Future Research" that we do not look at variation in perceived gravity within a scenario, but between scenarios. We clearly state here that this design decision may represent a weakness in terms of causality of results. Here we also address various other points of our design decision, which in retrospect may be considered not ideal.

We appreciate your time and effort and would like to thank the editor and reviewers for their suggestions which have helped us improve our work. We are glad to see how our manuscript has developed and hope that you find it suitable for publication. Should you find our paper in need of additional correction, we are of course prepared to make further changes.
